# Occupation as a risk factor of small cell lung cancer

**DOI:** 10.1038/s41598-023-31991-0

**Published:** 2023-03-23

**Authors:** Teresa Curiel García, Alberto Ruano-Ravina, Cristina Candal-Pedreira, Rafael López-López, María Torres-Durán, José Ramón Enjo-Barreiro, Mariano Provencio, Isaura Parente-Lamelas, Iria Vidal-García, Cristina Martínez, Jesús Hernández-Hernández, Ihab Abdulkader-Nallib, Olalla Castro-Añón, María Piñeiro-Lamas, Leonor Varela-Lema, Alberto Fernández-Villar, Juan Barros-Dios, Mónica Pérez-Ríos

**Affiliations:** 1 Service of Medical Oncology, Santiago de Compostela University Hospital, Santiago de Compostela, Spain; 2 Departamento de Medicina Preventiva y Salud Pública, Universidad de Medicina, Universidad de Santiago de Compostela, C/ San Francisco s/n., 15782 Santiago de Compostela, Spain; 3 Consortium for Biomedical Research in Epidemiology and Public Health (CIBER en Epidemiología y Salud Pública-CIBERESP), Madrid, Spain; 4 Health Research Institute of Santiago de Compostela (Instituto de Investigación Sanitaria de Santiago de Compostela-IDIS), Santiago de Compostela, Spain; 5 Translational Medical Oncology Group (Oncomet), Roche-Chus Joint Unit, Santiago de Compostela, Spain; 6 NeumoVigo I+i Research Group, Department of Pneumology, Alvaro Cunqueiro University Teaching Hospital, Southern Galician Institute of Health Research (Instituto de Investigación Sanitaria Galicia Sur-IISGS), Vigo, Spain; 7 Service of Medical Oncology, Puerta del Hierro University Hospital of Madrid, Madrid, Spain; 8 Service of Neumology, University Hospital Complex of Ourense, Ourense, Spain; 9 Service of Neumology, University Hospital Complex of A Coruña, A Coruña, Spain; 10 National Institute of Silicosis, University Hospital of Asturias, Oviedo, Spain; 11 Service of Neumology, Hospital Complex of Ávila, Ávila, Spain; 12 Service of Pathological Anatomy, University Hospital of Santiago de Compostela, A Coruña, Spain; 13 Department of Pneumology, Lucus Augusti University Teaching Hospital, Lugo, Spain; 14 Grupo C039 Biodiscovery HULA-USC, Health Research Institute of Santiago de Compostela, Santiago de Compostela, Spain

**Keywords:** Lung cancer, Small-cell lung cancer, Risk factors

## Abstract

Small cell lung cancer (SCLC) comprises approximately 10% of all lung cancer cases. Tobacco is its main risk factor; however, occupation might play a role in this specific lung cancer subtype. The effect of occupation on SCLC risk has been hardly studied and therefore we aim to assess the role of occupation on the risk of SCLC. To do this, we designed a multicentric, hospital-based, case–control study. Cases consisted exclusively in SCLC patients and controls were recruited from patients having minor surgery at the participating hospitals. Face to face interviews emphasizing occupation and tobacco consumption were held and residential radon was also measured. Logistic regression models were adjusted with odds ratios with 95%CI as estimations of the effect. 423 cases and 905 controls were included. Smoking prevalence was higher in cases compared to controls. Those who worked in known-risk occupations for lung cancer showed an OR of 2.17 (95%CI 1.33; 3.52), with a similar risk when men were analysed separately. The results were adjusted by age, sex, smoking and indoor radon exposure. Those who worked in known-risk occupations and were moderate or heavy smokers had a SCLC risk of 12.19 (95%CI 5.68–26.38) compared with never or moderate smokers who had not worked in such occupations. Occupation is a relevant risk factor of SCLC, and it seems that its effect is boosted when tobacco smoking is present.

## Introduction

Lung cancer is an important public health problem and the deadliest cancer in the world^1^. Five-year survival is around 20% despite recent advances in screening and new treatments^2^. This cancer is usually classified in two different types according to histology: Non-small cell lung cancer (NSCLC) and Small Cell Lung Cancer (SCLC) based on tumour origin, biological characteristics, and response to treatment (mainly chemotherapy). SCLC comprised approximately 10% of all lung cancers in 2019^2^ and has a worse survival compared to NSCLC, ranging from 2 to 10% at five years from diagnosis^3^, depending on cancer stage at diagnosis.

The most important risk factor of lung cancer is tobacco consumption, followed by indoor radon exposure^4,5^. This remains true when analysing SCLC specifically, though tobacco consumption is even more closely associated with SCLC than with other histological types. Practically all SCLC cases are ever smokers, but SCLC may appear in never-smokers^6^.

Occupation is a relevant risk factor of lung cancer and plays a very relevant role in those individuals who hold lung cancer risk occupations for several years. Exposure to carcinogens such as chromium, nickel, silica dust, beryllium, arsenic compounds or asbestos may be high in certain occupations (i.e. coke oven workers., road and railroad workers, miners, and so on). The most known occupations associated with lung cancer are those related with asbestos exposure (insulators, roofers, shipyards, etc.), but many other occupations have been classified as having an increased risk of lung cancer compared with the general population^7–9^. These occupations are extremely diverse and the number of employees in each one is highly variable. These are mostly occupations related with exposure to diesel exhaust, construction, wood working, heavy industry, ceramics, mining activities, stone processing, and many others. Of note, some studies have shown an interaction between the number of years worked in risk occupations of lung cancer and tobacco consumption, along with studies performed exclusively in workers exposed to asbestos^10,11^. Environmental (non-occupational) exposure to asbestos has also been associated with an increased risk of lung cancer^12^. There is evidence showing that those who have worked for more than 30 years in risk occupations of lung cancer and are heavy smokers might have a lung cancer risk higher than 275 compared to never smokers who have not worked in risk occupations^10^.

Despite this, and because the incidence of SCLC is low^2^, the availability of studies analysing specifically the role of occupation on the onset of SCLC is scarce, and usually comes from sub-analyses of studies including a majority of NSCLC cases. To our knowledge, there is no study that has focused only on SCLC, but many have analysed the risk of lung cancer associated with different occupational exposures and some have differentiated by histological subtype. These studies have found significant associations between SCLC and occupational exposure to polycyclic aromatic hydrocarbons^13^, crystalline silica^14^, diesel engine exhaust^15^ and asbestos^16^.

The main objective of this research is to ascertain the role of occupation on SCLC risk. As a secondary objective, we aimed to analyse if tobacco consumption might modify the risk of SCLC entailed by occupation in a multicentric hospital-based case–control study which only included SCLC cases.

## Subjects and methods

### Design and setting

We designed a multicentric, hospital-based case–control study. There were 11 recruiting hospitals from four Spanish regions: Galicia, Madrid, Castile-León and Asturias. All cases had to present an incident SCLC and in no case more than one month elapsed since histological confirmation to recruitment. In some hospitals the Service of Pathology provided the diagnoses and in others it was the oncologist who recruited the patient after having the pathologic report. The ICD-O-3 (International Classification of Diseases for Oncology, third edition) considered was C34 (morphology codes: 80413, 80423, -80433, 80443 and 80453). Cases and controls were recruited from September 2015 to August 2019. The study area is characterized as presenting high indoor radon concentrations^17^ and all population has universal healthcare coverage. The study protocol was approved by the Ethics Committee of the Santiago de Compostela-Lugo healthcare area (reference of approval 2015/222), and all participants gave written informed consent before being included. The study was competitively funded by the Instituto de Salud Carlos III (PI15/01211-Cofinanced FEDER) and by the Spanish Society of Neumology (ref 2022/1215).

### Inclusion and exclusion criteria

All participants had to be older than 30 years with no upper age limit and with no previous cancer diagnosed. Controls were selected at different hospitals from individuals who had undergone non-complicated or complex surgery. The main causes for hospital admission for controls were inguinal hernias, minor surgery in upper or lower limbs or lipomas. Controls were selected using a frequency-based sampling on age and sex regarding cases in order to have comparability for these two variables between cases and controls. Controls were recruited in parallel to cases.

### Information retrieval

Three information sources were used for all participants: a questionnaire, a radon device put in place in all participants’ dwellings and 3 ml of total blood (not used for this study). We used a semi-structured questionnaire which was fulfilled through a personal interview performed by trained nurses or clinical staff. It collected detailed information on lifestyle aspects and risk factors of lung cancer. All participants were asked for information regarding their tobacco consumption, age at initiation, current smoking status, number of daily cigarettes or other tobacco products smoked, and also if they had lived with smokers or not for the last 20 years. Detailed information was ascertained regarding their occupational history in a specific section of the questionnaire. They were asked for their three last occupations, and the number of years spent in each of them. We also asked for the number of hours spent daily on each of these occupations but we did not use them since having part-time jobs was unusual in the recruited sample. They were also asked regarding the specific role held in each occupation and on the exposure to different substances used in each of those occupations.

Radon information was obtained from each participant. A radon device was given at recruitment and was placed at the measured dwelling for at least three months. The device was of the alpha track type. Once that the measurement period finished and after receiving a phone call from the research team, the detector was sent back and read at an accredited facility with a long experience in radon measurements (Galician Radon Laboratory, University of Santiago de Compostela, https://www.radon.gal). All participants received information regarding their radon concentration at their dwellings (in becquerels per cubic metre).

### Data preparation for analysis

Industries and occupations were coded blindly with respect to case or control status by two of the authors with experience in epidemiology and oncology. Codes were translated into occupations with known (list A) or suspected (list B) carcinogenic risk to the lung^18^. Subjects with occupations from both lists were assigned to list A. This classification has been widely used for studying occupations linked to lung cancer because it is tailored for this disease. Lists A and B come from both International Standard Classification of Occupations (ISCO) and the International Standard Industrial Classification (ISIC), which has been widely used in most countries worldwide. Occupations present in list A comprise: vineyard workers; mining and quarrying; chemical industry; pesticide and herbicide production; asbestos production or remotion; metals (iron and steel industries); other metals industries (alloying, smelting, refining, etc.); shipbuilding, motor vehicle, railroad equipment manufacturing; coke plant workers and gas production workers; insulators and pipe coverers, roofers, asphalt workers; painters (construction and automotive industry). The reference group included subjects never employed in occupations present on either list. For each occupation entailing a known risk we added up the number of years, creating a continuous variable named “years worked in risk occupations of lung cancer”. Tobacco consumption was also transformed in a continuous variable creating the number of pack-years and afterwards dividing it into three categories: never-smokers, moderate-smokers and heavy-smokers. To do this, we divided smokers in two categories using the median number of pack years as the cutpoint.

### Statistical analysis

We first performed a bivariate analysis comparing SCLC cases characteristics with those of controls (age, sex distribution, differences in tobacco consumption, indoor radon exposure). A logistic regression using the case–control status was performed to show the risk of SCLC due to having worked in risk occupations known to present an excess risk of lung cancer (list A) or not. All participants were used, with those not having worked in list A occupations used as reference category (not exposed). This regression was adjusted by age (continuous), sex, tobacco consumption (continuous variable in pack-years) and indoor radon exposure (continuous variable). Indoor radon exposure was introduced as a continuous variable to favour the adjustment process and because the action level is not homogeneous between countries or international bodies. A second regression was performed using in this case as the main independent variable the number of years worked in known risk occupations of lung cancer (duration of work in risk occupations). This analysis was stratified by tobacco consumption through creating two categories: never smokers and moderate/heavy smokers, where risks due to years of occupation were calculated separately. The adjustment variables were the same as those used in the previous regression. All results are represented as Odds Ratios (OR) with their 95% confidence intervals (95% IC). The analyses were performed with R v.4.1.2 (R Core Team: The R project for statistical computing. R Found. Stat. Comput. 2021. https://www.r-project.org/).

### Ethical considerations

This study was implemented following good practice guidelines and the Declaration of Helsinki. The study protocol was approved by the Ethics Committee of the Santiago de Compostela-Lugo healthcare area (reference of approval 2015/222). An informed consent was obtained from all the participants.

## Results

The sample size included 423 SCLC cases and 905 controls, a total of 1328 participants. 26.7% and 32.9% of cases and controls were women, respectively. A sample description is shown in Table 1 broken down by case–control status. Cases were slightly older than controls. Tobacco consumption was much more frequent in cases; 91.6% were ever smokers compared to 56.8% in controls. The number of years with active working life was very similar (30 years for both groups of participants). The mean number of occupations for cases and controls were 1.3 and 1.4 for cases and controls, respectively, and 8.1% of cases spent more than 20 years in known-risk professions for lung cancer compared to 6.2% of controls. The most frequent known-risk occupations for participants were metal workers (35.8%), painters (21.2%) and stonemasons (13.9%).

**Table 1:** Sample description broken down by case–control status.

Variables	Cases, n (%)	Controls, n (%)
Number of participants	423 (31.9)	905 (68.1)
Age (years)		
Mean (95% CI)	65.7 (64.8–66.6)	60.2 (59.4–61.0)
Median (25–75th percentiles)	66 (59–72)	61 (53–68)
Sex		
Women	113 (26.7)	298 (32.9)
Men	310 (73.3)	607 (67.1)
Education^a^		
No formal studies	49 (12.1)	27 (3)
Primary school	228 (56.2)	488 (54.6)
High school	88 (21.7)	230 (25.7)
University degree	41 (10.1)	149 (16.7)
Number of years of active working life		
Mean (95% CI)	30.6 (28.9–32.4)	30.3 (29.3–31.4)
Median (25–75th percentiles)	33 (20–40)	32 (20–40)
Number of years spent in high-risk professions (> 1 year)		
Mean (95% CI)	24.7 (19.3–30.1)	23.1 (19.3–26.9)
Median (25–75th percentiles)	23 (12.5—37)	24.5 (7.3–36.8)
Subjects with over 20 years in a high-risk profession	17 (8.1)	33 (6.2)
Tobacco consumption^b^		
Never-smokers	33 (8.4)	382 (43.2)
Moderate smokers (1–37 pack-years)	90 (23.0)	338 (38.2)
Heavy smokers (≥ 38 pack-years)	269 (68.6)	165 (18.6)
Residential radon exposure Bq/m^3^		
Geometric mean (95% CI)	149.4 (136.4; 163.7)	154.7 (145.7; 164.1)
Median (25–75th percentiles)	140 (83; 251.8)	143 (90; 271.8)

^a^Missing data for 17 cases and 11 controls.

^b^Missing data for 31 cases and 130 controls.

When we analysed the association with the appearance of SCLC, we observed that participants who had ever worked in high-risk professions presented an unadjusted OR of 1.77 (95%CI 1.20–2.59). When other covariates were included (age, tobacco consumption and indoor radon exposure), the OR was 2.17 (95%CI 1.33–3.52) (Table 2). This result was very similar when the analysis was restricted to men (adjusted OR 2.09; 95%CI 1.28–3.40). For women, there were only four who worked in high-risk professions for more than 1 year. Therefore, the low sample size in this group did not allow the statistical adjustment of these results.

**Table 2:** Risk of small cell lung cancer for participants having worked in high-risk professions of lung cancer.

High risk occupations (list A)	Cases	Controls	OR^a^ (95% CI)
Global			
No	272	690	1 (–)
Yes	45	59	2.17 (1.33; 3.52)
Men			
No	195	440	1 (–)
Yes	43	57	2.09 (1.28; 3.4)
Women			
No	77	250	1 (–)
Yes	2	2	2.52 (0.17; 28.83)

^a^Adjusted by age, tobacco consumption and indoor radon exposure.

We analysed if the association posed by having worked in high-risk occupations was modified by tobacco consumption. These results are shown in Table 3. When never-smokers who never worked in high-risk occupations were considered as the reference category, we observed that ever-smokers had a higher lung cancer risk if they had worked in high-occupations for more than 10 years (OR: 12.19; 95%CI 5.68–26.38), compared with those not having worked in high-risk occupations (OR: 8.22; 95%CI 5.34–13.11). These results were adjusted for sex and indoor radon exposure. Men and women could not be analysed separately because there were no never smoking men among SCLC cases and there were no women having worked in high-risk professions.

**Table 3:** Small cell lung cancer risk stratified by tobacco consumption.

Variables^a^	Cases	Controls	OR (95% CI)^b^
Global			
Never smokers			
0 years	27	310	1 (–)
1–10 years in high-risk professions	1	6	2.28 (0.12; 14.25)
> 10 years in high-risk professions	0	13	0 (0; 0)
Moderate and heavy smokers			
0 years	245	380	8.22 (5.34; 13.11)
1–10 years in high-risk professions	5	12	5.58 (1.65; 16.70)
> 10 years in high-risk professions	19	21	12.19 (5.68; 26.38)

^a^Missing information for 126 cases and 163 controls.

^b^Adjusted by sex and indoor radon exposure.

## Discussion

This study shows that occupation entails a risk of developing SCLC. To our knowledge, this is one of the few studies exclusively recruiting SCLC cases for this purpose. For those having worked in risk occupations for lung cancer, SCLC risk suffered a twofold increase compared with those participants who have not worked in such occupations. Tobacco consumption seems to increase lung cancer risk for those sharing tobacco habit and risk occupations compared with smokers who have not worked in such occupations. Unfortunately, the number of women included and the low number of never smokers among cases limit the validity of our results.

It is well known that occupation is an important risk factor for lung cancer. In fact, exposure to carcinogens at work should be avoided in order to reduce the cancer risk, according to the latest version of the European Code against Cancer^19^. A number of monographies published by the International Agency for Research on Cancer have recognized that many occupations may increase the risk of lung cancer^20,21^. This is the case of those who are exposed to asbestos, silica dust, professional drivers or exposed to diesel exhaust, mining activities (gamma radiation, radon gas), and works related with construction (painters, carpenters, joiners) or heavy industry (coke oven workers, ceramics, among others).

Despite this evidence, it is extremely difficult to assess the role of specific occupations on lung cancer risk because of the following problems: (a) people may change frequently from one occupation to another, (b) even in the same occupation, the degree of exposure to carcinogens may vary, due to the different availability of protection equipment, awareness of the worker, and specific activity developed by the worker, (c) even for workers who have worked in the same occupation for years, lung cancer may be developed many years after retirement, making difficult to establish an association with previous occupations and, (d) the role of tobacco consumption is a very relevant confounding variable on the real risk of certain occupations. It is important to mention that manual workers (called sometimes blue-collar workers) usually tend to smoke with a higher frequency and at a higher amount that their counterparts (white-collar or office workers)^22^. Therefore, it is difficult to disentangle the real role of occupation on lung cancer risk when there is a really important risk factor such as tobacco^23^.

In this research we aimed to ascertain if occupation was also a risk factor for SCLC. SCLC is the lung cancer type entailing the highest relationship with tobacco consumption, and therefore most SCLC are heavy smokers. This aspect may have partly blurred the association between occupation and SCLC risk. In our sample, 68.6% of SCLC cases smoked more than 38 pack-years (a quantity close to 1 pack per day for 40 years), compared to an equivalent figure of 18.6% among controls.

Other studies have analysed the role of occupations known to present an excess risk of lung cancer, but we are not aware of any study specifically designed to assess SCLC risk. Those studies showing results for SCLC patients have observed that workers on occupations known to be associated with lung cancer have an increased risk of this cancer type^24,25^.

The molecular mechanisms implicated on the appearance of SCLC are unclear and may be different by each of the occupations analysed. For example, those occupations where radon plays a role could present different molecular fingerprints (as shown in previous studies)^26^ than in those where asbestos exposure or silica dust appear as the main carcinogens. The molecular pathways of asbestos or silica dust causing lung cancer is not fully understood, and some genes have been proposed to be involved mainly in mesothelioma and non-SCLC^27,28^, but we are not aware of any study specifically addressing SCLC carcinogenesis due to asbestos or silica dust exposure.

This research presents important limitations. The main one is its limited sample size coupled with the large number of different occupations held by participants during their lifetime. This fact has not allowed us to analyse each specific occupation separately. This limitation is also related to the lists we have used. These lists classify occupations as those posing a clear excess of lung cancer (list A) and those with a suspected risk (list B). We only used participants who have been employed in list A classifications because using list B could mean to mix a confirmed risk occupation with a suspected risk occupation and perhaps this could bias the results to the null hypothesis. A further limitation is that there was a very low number of never smokers and therefore using this category as a reference to formally analysing the potential interaction (additive, multiplicative) between tobacco consumption and occupation was not possible. The low number of women working in high-risk occupations for lung cancer is also a further limitation. This is because most Spanish women aged 60 or more use to be housewives or in some cases perform agricultural work.

The present study has some fortresses. The main one is that it consists in a multicentric study where 11 hospitals recruited patients, increasing its external validity. This is also the first study analysing specifically SCLC patients and was designed to include only this type of lung cancer. Furthermore, it has adjusted the results by indoor radon exposure, which is recognized as the main risk factor of lung cancer in never smokers and the second after tobacco consumption in ever-smokers by WHO and USEPA^4^. Previous studies have also related this indoor carcinogen with the appearance of SCLC^26,29^.

To conclude, occupation is a risk factor of SCLC, though at a lower extent than tobacco consumption. This risk is present for those who have worked for more than 10 years in such occupations. More studies are needed on this histological type to assess the role of specific occupations and also in women, in order to know how occupation may increase their risk of SCLC. Any worker in a high-risk occupation for lung cancer must know that if he or she smokes lung cancer risk increases importantly, and therefore these workers should be approached by prevention services to promote tobacco cessation activities.

## Data Availability

The datasets generated during and/or analysed during the current study are available from the corresponding author on reasonable request.
